# Evaluation of the Covalent Functionalization of Carbon Nano-Onions with Pyrene Moieties for Supercapacitor Applications

**DOI:** 10.3390/ma13051141

**Published:** 2020-03-04

**Authors:** José D. Velásquez, Monika Tomczykowa, Marta E. Plonska-Brzezinska, Manuel N. Chaur

**Affiliations:** 1 Departamento de Química, Facultad de Ciencias Naturales y Exactas, Universidad del Valle, 760001 Cali, Colombia; jose.velasquez.carmona@correounivalle.edu.co; 2 Department of Organic Chemistry, Faculty of Pharmacy with the Division of Laboratory Medicine, Medical University of Bialystok, 15-222 Bialystok, Poland; monika.tomczyk@umb.edu.pl; 3 Centro de Excelencia en Nuevos Materiales (CENM), Universidad del Valle, 760001 Cali, Colombia

**Keywords:** carbon nano-onion, carbon nanostructure, supercapacitance, faradaic capacitance, π–π stacking, pyrene moieties

## Abstract

Herein, we report the surface functionalization of carbon nano-onions (CNOs) through an amidation reaction that occurs between the oxidized CNOs and 4-(pyren-4-yl)butanehydrazide. Raman and Fourier transform infrared spectroscopy methods were used to confirm the covalent functionalization. The percentage or number of groups in the outer shell was estimated with thermal gravimetric analysis. Finally, the potential applications of the functionalized CNOs as electrode materials in supercapacitors were evaluated by cyclic voltammetry and electrochemical impedance spectroscopy. Functionalization increased the specific capacitance by approximately 138% in comparison to that of the pristine CNOs, while acid-mediated oxidation reduced the specific capacitance of the nanomaterial by 24%.

## 1. Introduction

The use of carbon nanomaterials (CNs) has been quite popular for the construction of supercapacitors (SCs) [1,2,3,4,5], specifically in electrochemical double-layer capacitors (EDCLs) [6,7]. Carbon nanotubes (CNTs) and graphene shells are frequently used for energy storage, commonly as a hybrid metal type material [8,9,10]. The energy storage mechanism is based on the adsorption of electrolyte ions over a large specific surface area of electrically conductive porous electrodes [11,12]. Therefore, properties such as high conductivity, large surface area, corrosion resistance, temperature stability, and porosity are considered key characteristics for the development of these devices [13] Active and porous carbon compounds, such as carbide-derived carbons, templated carbons, graphene nanotubes, and carbon nanotubes, are the most commonly investigated materials for energy storage applications [14]. Likewise, multilayer concentric fullerenes, denoted as carbon nano-onions (CNOs), which were discovered by Iijima in 1980 [15], are promising CNs for SC applications because of their intrinsic properties of high mesoporosities and large surface areas [11,16,17,18,19]. It is well-known that the functionalization of the outer surface by different routes improves the process in which CNOs interact with electrolytes [20,21,22,23,24,25]. In this regard, the capacitive power of an SC is largely associated with an optimal electrode/electrolyte interface, where changes in morphology (rugosity, porosity, channels, etc.) are due to intermolecular forces and/or intra-interclusters, which play a major role [26,27,28,29,30]. Functionalizing with the correct structures that can enhance power density by means of interactions with electrolytes or even between nanoparticles is a challenge in this field [29]. Condensed benzene ring molecules can create π–π stacking interactions that could improve the performance of CNOs in two ways: (i) by using non-aqueous electrolytes, such as planar ionic liquids, which can work in a wide operating voltage window; and (ii) by favoring a regular porosity with narrow pore size distributions. The latter, according to Konrad and coworkers, who studied the concept of the “best porous size,” is considered the most important characteristic in the mechanistic process of charging and discharging in SCs [31]. 

In contrast with the investigation done by Giordani et al., who applied non-covalent functionalization to pyrene-BODIPY (boron-dipyrromethene) dyads for biological imaging [32], herein, we report the first covalent functionalization of CNOs with hydrazyl pyrene groups to modify the morphology of the CNO surfaces and therefore analyze the effect of functionalization on the specific capacitance. This study demonstrates that functionalization with pyrene moieties, such as the model of condensed rings, is an effective method for improving the capacitance of CNO electrodes when compared to the capacitance of pristine CNOs (*p*-CNOs).

## 2. Materials and Methods

### 2.1. Synthesis of p-CNOs

Ultradispersed carbon nanodiamonds (NDs) (Sigma-Aldrich, St. Louis, MO, USA) with 10 nm average diameters were annealed in a Nabertherm LHT 02/18 oven (Lilienthal, Germany) at 1600 °C for 2 h under an inert atmosphere of nitrogen. The final temperature was maintained for 1 h, and then the material was slowly cooled to room temperature over a period of 1 h. Then, the residual amorphous carbon was eliminated by heating at 400 °C under air for 2 h. The final product was the pristine CNOs.

### 2.2. Functionalization of the p-CNOs

First, 40 mg of the *p*-CNOs were oxidized with a mixture of HNO_3_/H_2_SO_4_ (1:3) (15 mL under reflux) for 1 h. Afterward, 1 M NaOH was used to neutralize the solution. The precipitate was washed with water and methanol by centrifugation. The solid was vacuum dried for 12 h at 60 °C to yield the oxidized carbon nano-onions (*ox*-CNOs). Thereafter, 20 mg of the *ox*-CNOs were dispersed under sonication in chloroform (10 min), and then 500 mg of 4-(pyrene-4-yl)butanehydrazide, which was previously dissolved in chloroform, and 500 µL of triethylamine (TEA) (used as a base catalyst in the amidation process) were mixed and refluxed for 5 days. The carbon nano-onions that were functionalized with the pyrene derivatives (*pyr*-CNOs) were washed by centrifugation with chloroform, methanol, and water and then dried for 24 h in a vacuum oven at 60 °C.

### 2.3. Methods

The *p*-CNOs were examined with a Libra 120 high-resolution transmission electron microscopy (HR-TEM) system (Zeiss, Germany). The accelerating voltage of the electron beam was 20–30 keV. A Shimadzu IR Affinity-1 instrument that was equipped with attenuated total reflectance (ATR) and a ThermoScientific DXR Smart Raman instrument (Waltham, MA, USA) that was equipped with a 532 nm laser beam were used for the Fourier transform infrared spectroscopy (FT-IR) and the Raman analysis, respectively. The morphologies without any coatings were studied by using a JEOL JCM-5000 NeoScope scanning electron microscopy (SEM) system (Tokyo, Japan). The carbon powder was deposited on a carbon film. A high vacuum and source power between 10 and 15 kW were used. Thermogravimetric analysis (TGA) and differential scanning calorimetry (DSC) were carried out by using a TGA/DSC Mettler Toledo system (Columbus, OH, USA) by heating in air from 100 to 900 °C with a rate of 10 °C/min. 

A three-electrode system was used for the cyclic voltammetry (CV) experiments, which used a Pt mesh as the counter electrode, Ag/AgCl as the reference electrode, a glassy carbon (GC) disk with a 3 mm diameter as the working electrode, and a 1 M Na_2_SO_4_ solution as the electrolyte. The carbon nanostructures were sonicated in methanol (1 mg/mL). A 10–20 μL drop of the solution that contained the dispersed functionalized or non-functionalized CNs was then deposited on the GC electrode surface. After the solvent evaporated, the electrode was introduced into the solution electrolyte. CV was performed with scan rates of 20, 30, 40, 50, 100, 200 and 500 mV/s in an electrochemical window of -30 to 800 mV vs. Ag/AgCl. The electrochemical impedance spectroscopy (EIS) spectra were obtained in a range from 100 mHz to 10 kHz, with 5 mV as the voltage amplitude.

## 3. Results and Discussion

### 3.1. Physicochemical Characterization of the CNOs 

**Figure 1:**
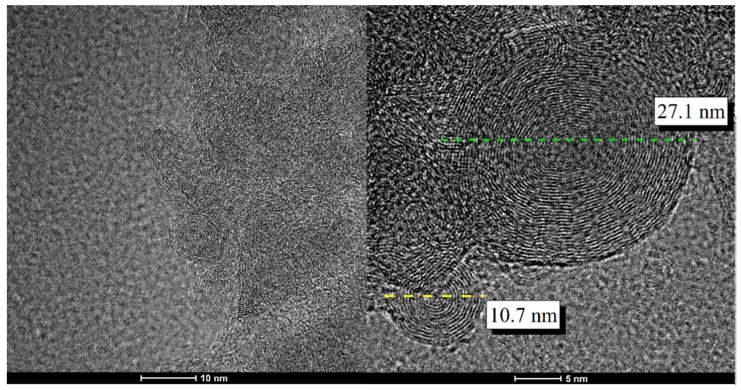
High-resolution transmission electron microscopy (HR-TEM) images of the pristine carbon nano-onions (*p*-CNOs) that were obtained by annealing of ultradispersed carbon nanodiamonds under a nitrogen atmosphere at 1600 °C.

High-resolution transmission electron microscopy (HR-TEM) showed the formation of spherical concentric shells, a characteristic feature of *p*-CNOs (Figure 1). The diameter of the carbon nanoparticles ranged from 5.0 to 30.0 nm. These nanostructures consisted of approximately six-to-ten graphenic shells with interlayer distances of 0.34 nm (based on the diffraction patterns), which was approximately equal to the distance between two graphitic planes (0.334 nm) [33]. The surface of *p*-CNOs is mainly composed of hexagonal rings that are similar to graphene layers but include some pentagonal rings, defects, and holes [34,35]. The structural defects and high sphericity lead to the relatively high chemical reactivity of *p*-CNOs.

**Scheme 1:**
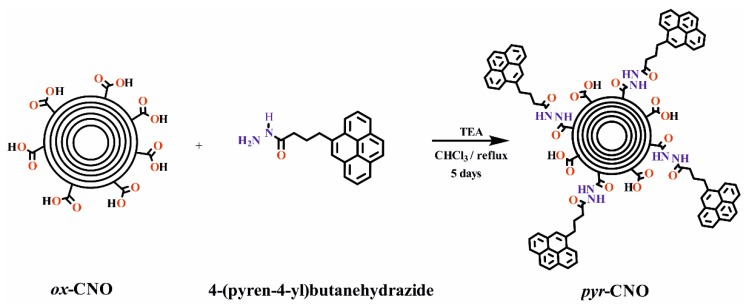
Functionalization of oxidized-CNOs (*ox*-CNOs) through amidation with pyrene hydrazide.

**Figure 2:**
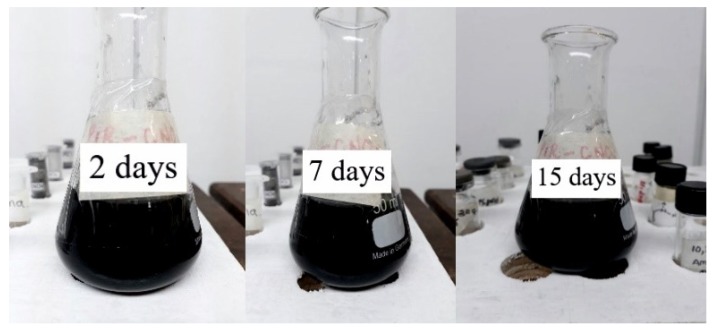
Dispersions of CNOs that were functionalized with the pyrene (*pyr*-CNOs) in water.

The *p*-CNOs underwent an acid treatment (HNO_3_/H_2_SO_4_; v/v = 1/3) to yield the *ox*-CNOs. Then, the reaction of the *ox*-CNOs with 4-(pyrene-4-yl)butanehydrazide yielded *pyr*-CNOs (Scheme 1). As a first qualitative observation, the oxidation of the *p*-CNOs resulted in a marked improvement in their ability to disperse in water, suggesting the presence of carboxylic groups on the surface of the CNOs. Likewise, aqueous dispersions of *pyr*-CNOs were very stable (no sedimentation was observed), even for weeks (Figure 2), indicating their potential for use as electrode materials, since homogeneity is an important factor when constructing SCs [36].

**Figure 3:**
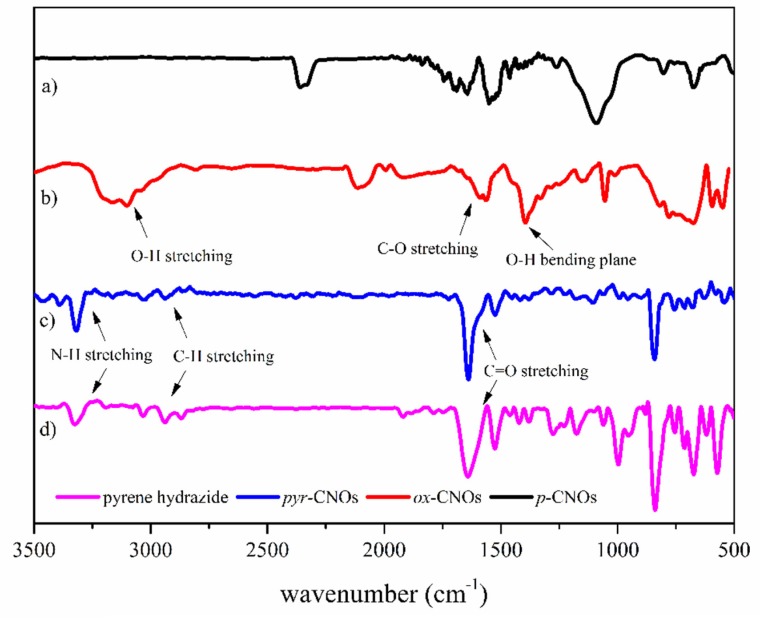
FT-IR spectra of (**a**) *p*-CNOs, (**b**) *ox*-CNOs, (**c**) *pyr*-CNOs, (**d**) pyrene hydrazide.

The Fourier transform infrared spectra (FT-IR) showed the presence of carboxylic and pyrene groups on the functionalized CNOs (Figure 3). Based on IR spectroscopy, a broad absorption band that extended from 2800 to 3200 cm^−1^ indicated the presence of carboxylic groups (stretching vibrations of O–H) [37]. The intense absorption bands at 1390 and 1570 cm^−1^ were due to the in-plane bending vibrations of O–H and the stretching vibrations of C–O, confirming the oxidation of *p*-CNOs to *ox*-CNOs (Figure 3b), which is similar to other studies [37,38]. The spectral features between 1540 and 1590 cm^−1^ may have also been related to carboxyl and carbonyl groups [39]. The stretching of the C=O and N–H bonds at 1625 and 3315 cm^−1^, respectively, proved the presence of pyrenic moieties on the CNO surface (Figure 3c). Other bands with lower intensities are observed at between 2800 and 2940 cm^−1^. These less intense bands are associated with the symmetric and asymmetric stretching of C–H bonds.

**Figure 4:**
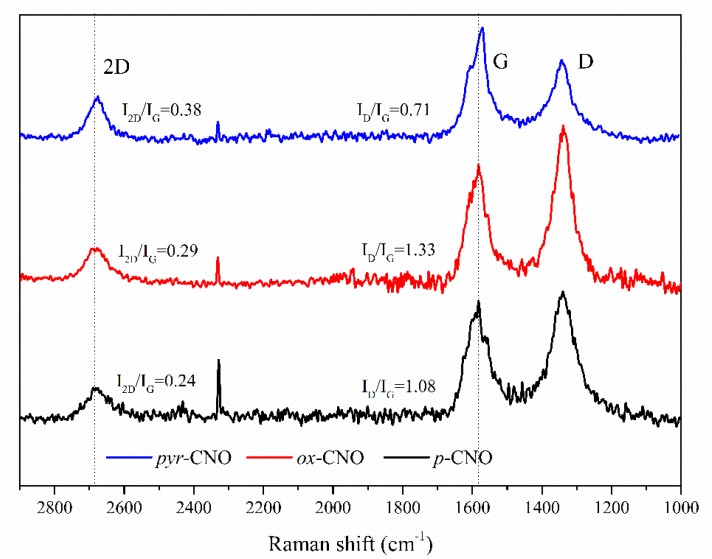
Raman spectra of the functionalized and non-functionalized CNOs, as obtained with 532 nm laser excitation.

The Raman spectra (Figure 4) show the characteristic G and D bands of the functionalized and non-functionalized CNOs. These bands were related to the graphitization degree or C atoms with *sp^2^* hybridization (G band) and the disorder degree or C atoms with *sp^3^* hybridization (D band). Likewise, a second-order band, 2D or G’, was also observed [40]. The ratio of the intensities of the D and G bands (I_D_/I_G_) confirmed the covalent functionalization process (Table 1). Additionally, the D and G lines were intense and broad, and the D band was more intense than the G band. In this respect, our spectra were similar to those of strongly disordered graphene [41].

Increases in the D band intensity in the Raman spectrum and in the I_D_/I_G_ ratio of *p*-CNOs and *ox*-CNOs were observed. Oxidation with strong acids caused the outer shell to rupture, and therefore, the hybridization of the C atoms changed to *sp^3^*, which was confirmed by the presence of oxygen groups (carboxylic, hydroxylic and carbonyl groups) [37]. The general equation reported by Cançado et al. was used to determine the crystallite size (*L*_a_) by Raman spectroscopy [42]:

(1) $$L_{a}=\left(2.4\times 10^{-10}\right)\lambda^{4}{\left(I_{D}/I_{G}\right)}^{-1}$$

where λ is the wavelength of the laser in nanometers (532 nm).

The intensity of the D band depends on the wavelength of the excitation light, which is due to the resonance Raman effect [43]. For our investigated materials, the TEM images showed that the smallest indivisible unit was the CNOs with diameters of approximately 5 nm (six-shell CNOs). Moreover, the spherical nanoparticles could easily aggregate. In the case of the CNOs, the I_D_/I_G_ ratio may provide information about the size and edges of the aggregates (crystallite sizes) [41]. In our investigations, we estimated the value defined in the literature as the average crystallite size *L_a_*, which was ca. 17.8 nm for the *p*-CNOs. After strong oxidation, the size decreased to approximately 14.5 nm. Taking into account all the known facts from the literature and our experimental data [37,44,45,46,47], we concluded that one aggregate could be formed with two or three CNO nanostructures.

**Figure 5:**
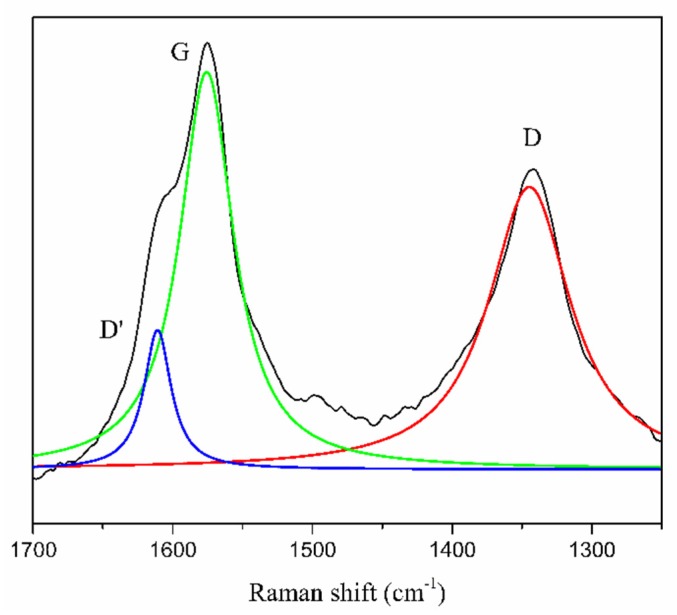
Deconvolution of the G and D bands in the *pyr*-CNOs Raman spectrum.

The G band of the *pyr*-CNO spectrum increased with respect to one of the *ox*-CNOs, showing an unusual and opposite behavior. This band is associated with the phonon E_2g_, which corresponds to the lateral vibration of the hexagonal condensed rings in the graphenic layers [48,49,50]. It is possible that this vibration was given by the pyrene structure; if so, the confirmation of covalent functionalization by using the ratio I_D_/I_G_ could not be conclusive. However, the G band in the *pyr*-CNOs spectrum exhibites a large full width at half maximum (FWHM) value. Additionally, the same spectrum shows an apparent shoulder that corresponds to the D’ band at ~1614 cm^−1^ (Figure 5). The latter is produced by the process of double resonance, which could be activated by the crystalline defects and could be associated with covalent functionalization [48].

**Figure 6:**
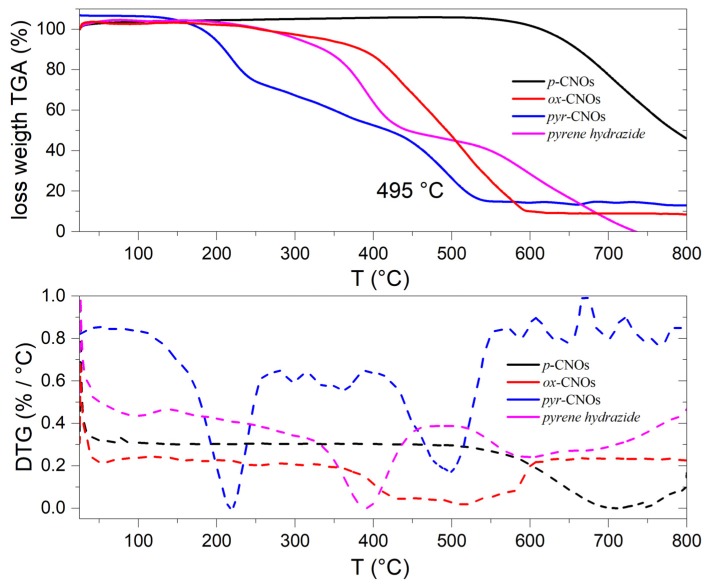
TGA curves of the functionalized and non-functionalized CNOs (**top**) and pyrene hydrazide under an air atmosphere. The first derivative of the corresponding TGA curve (**bottom**).

On the other hand, the thermogravimetric analysis (TGA) (Figure 6) showed that the *p*-CNOs (701 °C) had a higher decomposition temperature than the *ox*-CNOs (515 °C). This behavior can be attributed to the outer shell rupturing upon oxidation. The 20% loss of weight at 257 °C corresponded to the carboxylic fragments degrading in air. The TGA curve of the *pyr*-CNOs showed three decomposition temperatures that could correspond to: (i) non-amidized carboxylic groups at ~218 °C; (ii) covalent pyrene moieties that were functionalized at ~356 °C; and (iii) the carbon structure with a decomposition temperature of 495 °C.

By considering the average of eight fullerenic concentric layers (HR-TEM images) and employing an approximation that was previously used by Cioffi and coworkers [51], the number of groups attached to the outer shell was estimated. In this regard, the ox-CNOs contained ≈362 carboxylic fragments or 1 per every 10 carbons in the outer shell. On the other hand, the pyr-CNOs contained ≈306 pyrene moieties or 1 per every 13 carbons in the outer shell.

**Figure 7:**
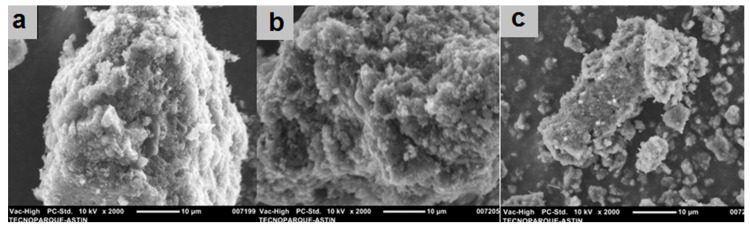
SEM images of the (**a**) *p*-CNOs, (**b**) *ox*-CNOs and (**c**) *pyr*-CNOs.

The scanning electron microscopy (SEM) studies of the three carbon nanomaterials are shown in Figure 7. The pristine and functionalized CNO surfaces exhibited a rough texture and porous morphologies, with many channels and three-dimensional aggregates, which may have contributed to improving the capacitive properties of the CNOs through the storage of the charge mechanism.

### 3.2. Electrochemical Properties of the CNOs and their Derivatives

**Figure 8:**
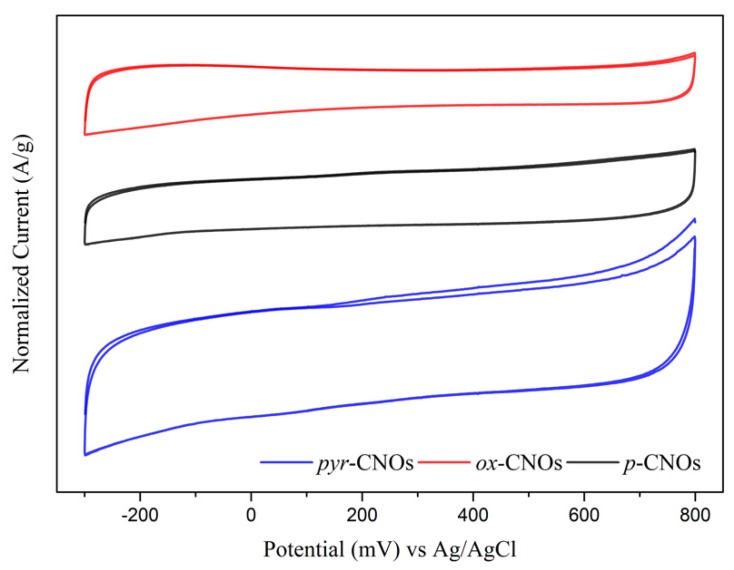
Cyclic voltammetry (CV) curves of the glassy carbon electrode that was covered with the functionalized and non-functionalized CNOs in 1.0 M Na_2_SO_4_, obtained with a sweep rate of 30 mV/s.

The cyclic voltammetry (CV) curves (Figure 8) show the characteristic pseudo-rectangular profiles of non-faradaic capacitive materials [52]. Therefore, the specific capacitance (*C_S_*) was determined based on Equation (1):

(2) $$C_{s}=\frac{\int_{E_{1}}^{E_{2}}i\left(E\right)dE}{2vm\left(E_{2}-E_{1}\right)}$$

where *v* is the scan rate, the numerator corresponds to the total charge obtained by the integration of the instant current with a scan rate *v*, *E*_2_ and *E*_1_ are the potentials of the electrochemical window, and *m* is the mass of the active material. The film exhibited a stable behavior under cyclic voltammetric conditions. No changes in the current were observed after prolonged potential cycling in the potential range from −0.30 to +0.80 V vs. Ag/AgCl. Our results showed that the acid-mediated oxidation of the CNOs decreased the capacitance, which was likely due to the decrease in the conductivity as a consequence of structural damage on the surface. On the other hand, the CV curve of the *pyr*-CNOs had a larger area, indicating an increase in the capacitance. This fact was explained by the larger porosity, which was due to the organized CNO agglomerates on the GC surface [53,54]. 

Table 2 summarizes the specific capacitances that were obtained for the *p*-CNOs, *ox*-CNOs and *pyr*-CNOs, which were calculated with a scan rate of 30 mV/s. The specific capacitance could also be calculated from Equation (2):

(3) $$i_{c}=C_{s}vm$$

where *i_c_* is the capacitive current, *v* is the scan rate, and *m* is the mass of the deposited material on the electrode.

**Figure 9:**
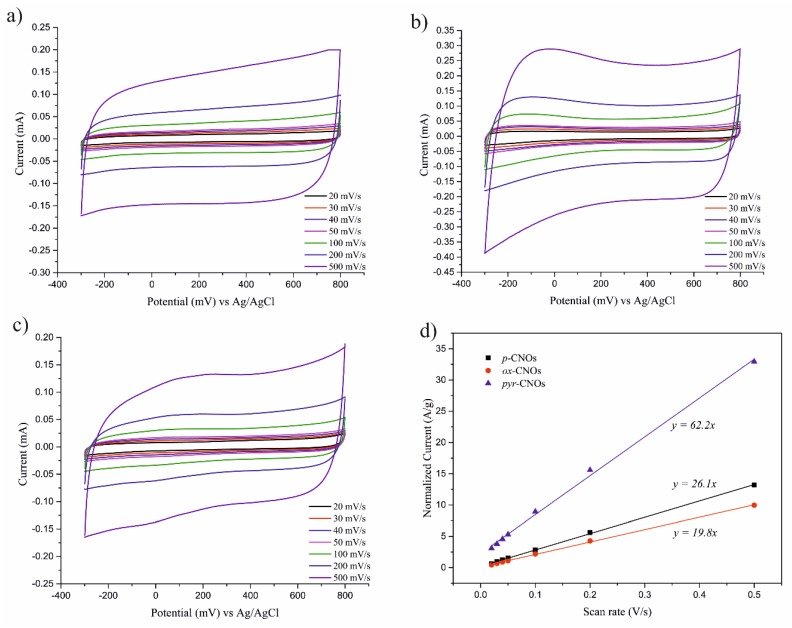
Dependence of the CV curves on the scan rate in the (**a**) *p*-CNOs, (**b**) *ox*-CNOs, and (**c**) *pyr*-CNOs. (**d**) Linear regression of the current density against the scan rate.

Given the existence of a linear correlation between the current peak and the scan rate, the specific capacitance could be calculated from the slope of the linear fit [55]. Figure 9 shows the CV curves that were obtained for each nanomaterial at 20, 30, 40, 50, 100, 200 and 500 mV/s, showing an increase in the current peak with increasing *v*. A linear relation between the capacitive current, *i_c_*, and the sweep rate, *v*, was observed (Figure 9d). From the slope of the *i_c_ – v* function, the specific capacitance of the *p*-CNOs, *ox*-CNOs and *pyr*-CNOs was found (Table 2). Despite the faradaic component, the film that contained the *ox*-CNOs showed a much lower capacitance than *p*-CNOs. Strong oxidation caused damage to the carbon structure, which affected the intrinsic properties of the CNOs [56]. Table 2 compares the specific capacitance values that were obtained by both methods. The *pyr*-CNOs had the highest specific capacitance value, which as equal to 62.2 F/g.

Table 3 summarizes some of the specific capacitance values of the CNO materials, as reported by other authors. As can be observed, CNOs have mainly been studied in composites with electroactive materials, such as metal cations, C_60_, polyaniline (PANI) and poly(3,4-ethylenedioxythiophene)-poly(styrenesulfonate) (PEDOT:PSS), in which some of them reported larger values than our study. Nevertheless, *pyr*-CNOs are a non-hybrid material that exhibits a high-intermediate value, which opens the door for its use in composites.

**Figure 10:**
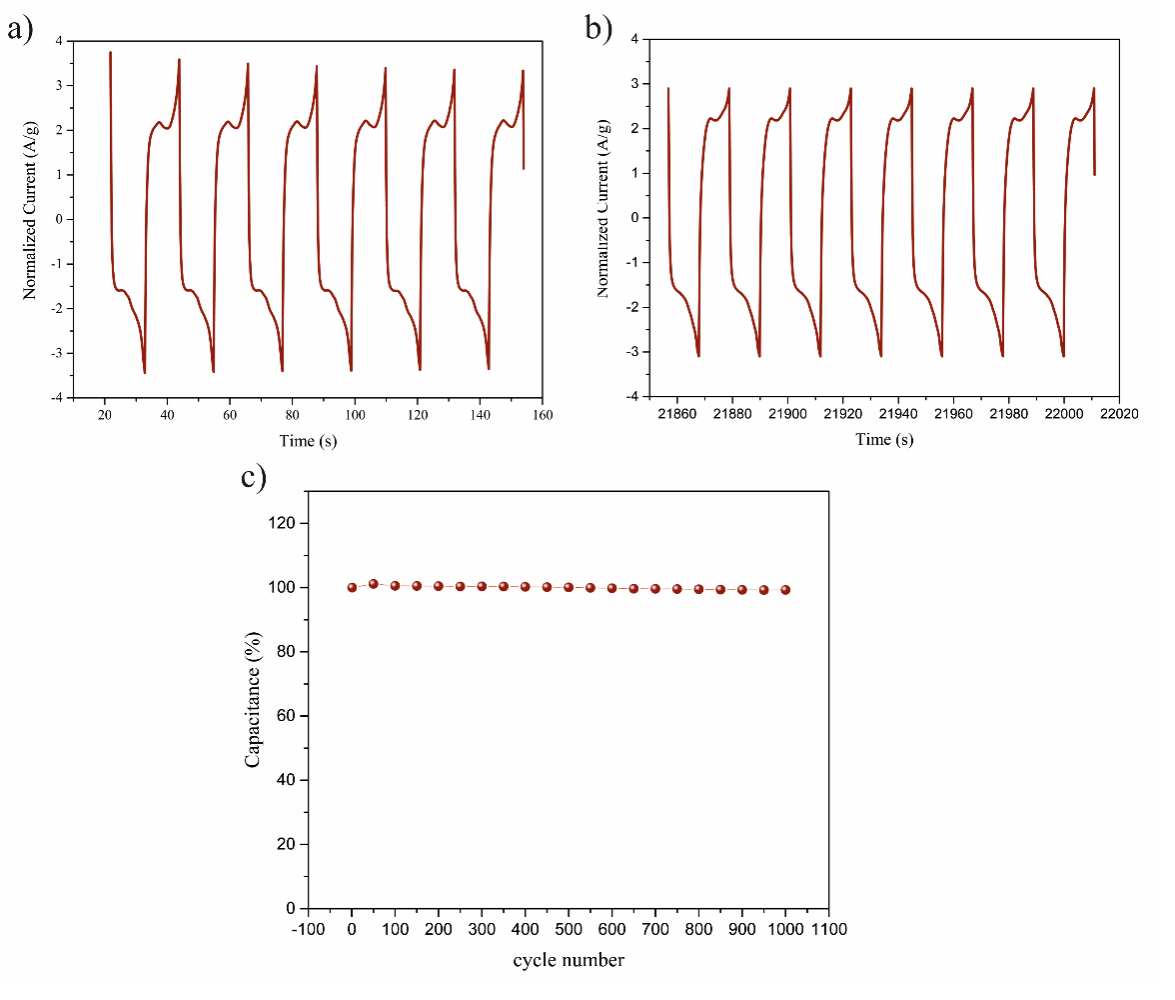
Stability studies of *pyr*-CNOs based on the CV measurements (1000 cycles): (**a**) the first 8 cycles, (**b**) the last 8 cycles, and (**c**) capacitive current values as a function of cycling numbers.

The long cycling stability performance (the mechanical and electrochemical stability) of an SC is a fundamental requirement for materials with applications in energy storage devices. It was found that the CVs for *pyr*-CNOs were stable in the 1 M Na_2_SO_4_ solution at 100 mV/s (Figure 10). Tests were conducted by using 1000 cycles, and the resulting capacitive currents as a function of cycling numbers are shown in Figure 10c. The *pyr*-CNO electrodes exhibited a long-term cycling stability, retaining approximately 99% of their initial capacitive current after 1000 cycles. Figure 10a,b shows the first and the last eight cycles of the CV measurements, respectively. The normalized current decreased from 3.7 to 2.9 A/g between the first and the last cycle.

Electrochemical impedance spectroscopy (EIS) is a technique that can provide information about the kinetics process of the electrolyte diffusing into the pores of the CNs, the effect of the resistance on the electron transfer and the contribution of each type of capacitance (EDLC or pseudocapacitance) [66]. The Nyquist plot and the Bode plot explain these results graphically, showing the relationship between the imaginary (Z’’) and the real (Z’) components of the impedance shown in the Nyquist plot, and the angle phase (ϕ) or impedance (Z) is plotted against the frequency in the Bode plot.

**Figure 11:**
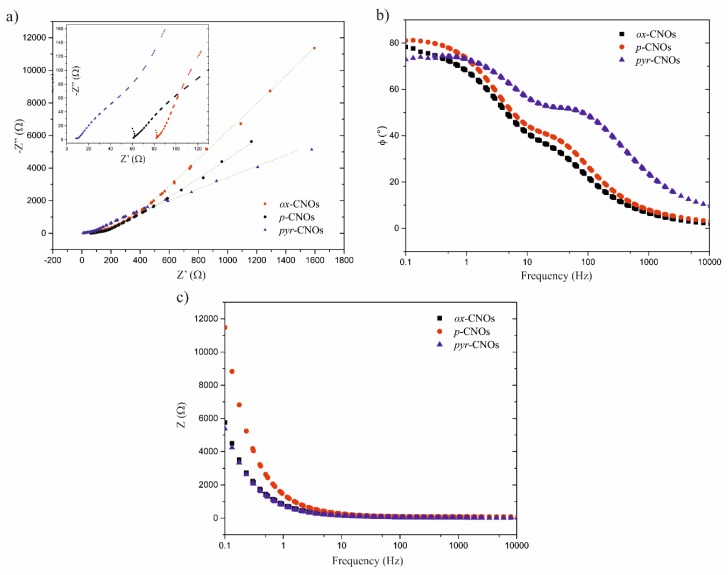
(**a**) Nyquist plot (Z’’ vs. Z’). (**b**) Bode plot (*ϕ* vs. *f*). (**c**) Bode plot (*Z* vs. *f*) obtained for the GCE that was modified with the CNOs.

**Scheme 2:**
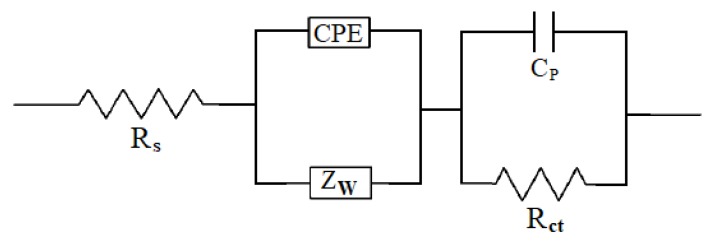
Equivalent electrical circuit representing the behavior of the CNOs in an aqueous electrolyte solution.

Figure 11a shows the EIS results that were obtained for the pristine and functionalized CNOs. The impedance spectra of the CN exhibited the shape that was expected for double-layer charging porous electrodes [13]. It is possible to identify two regions in the Nyquist plot: (i) the Knee frequency, which corresponds to the high-frequency region and is related to the beginning of the diffusion or charge-transfer process of the electrolyte at the electrode–electrolyte interface, and (ii) the Warburg finite-length diffusion at a low frequency, which is responsible for diffusion within the electrode [66,67]. The absence of a pronounced loop and the almost vertical line observed in the Nyquist plot at high and low frequencies, respectively, were useful to propose the equivalent circuit that describes the electrochemical behavior (Scheme 2).

The green line in Figure 11a shows a good fit between the experimental results and the proposed equivalent circuit. This equivalent circuit consists of a solution resistance (*R_s_*), the constant phase element (CPE) (which is related to the double-layer capacitance (*C_dl_*)), the pseudocapacitance (*C_p_*), the resistance at the electrode/nanomaterial interface (*R_ct_*), and the Warburg impedance (*Z_w_*). The latter represents the transport of counterions through the film during charging and is therefore evidence of the dependence on diffusion [68]. The electrical impedance is calculated by $1/Y_{o}{\left(j\omega \right)}^{n}$, where *Y_0_* is a constant related to the admittance and has dimensions of F. The *n* parameter can take values between 1 and 0, exhibiting an ideal capacitor behavior when *n* = 1 and a resistor behavior when *n* = 0. The parameters of each equivalent circuit component were obtained by the fitting procedure (see Table 4). In this sense, the lower *R_s_*, *R_ct_* and *Z_w_* values of the *pyr*-CNOs explained the increase in the specific capacitance upon functionalization and, therefore, the greater capacity to store energy. Even though the contribution of *C_dl_* was lower for the *pyr*-CNOs than for the *p*-CNOs, it was compensated for by the *C_p_* contribution. The *n* values that were obtained for our compounds were close to unity, indicating an almost ideal capacitive behavior.

The Bode plot of the phase angles vs. frequencies (Figure 11b) shows that the observed phase angles (*ϕ*) at a low frequency were 80.6°, 77.9° and 73.2° for the *p*-CNOs, *ox*-CNOs and *pyr*-CNOs, respectively. All the CNOs deviated from the ideal value of 90° at low frequencies, indicating the faradaic contributions to the charge storage mechanism [13]. In this regard, the electron density of the aromatic rings could lead to greater faradaic contributions and, consequently, a larger deviation in *ϕ* (73.2° vs. 90°) [69,70]. The Bode plot of the impedance vs. frequency (Figure 11c) also shows that the *pyr*-CNOs possessed a lower impedance than the *ox*-CNOs, including that the functionalization process that was performed with the pyrene groups could restore the intrinsic properties of the nanomaterial, despite the previous oxidation process.

The capacitance C(ω) can also be written in its complex form [71]:

(4) $$C\left(\omega \right)=C^{\prime }\left(\omega \right)-jC^{\prime \prime }\left(\omega \right)$$

where:

(5) $$C^{\prime }\left(w\right)=\frac{-Z\prime \prime \left(w\right)}{w\;\left|Z{\left(w\right)}^{2}\right|}$$

and:

(6) $$iC^{\prime \prime }\left(w\right)=\frac{Z^{\prime }\left(w\right)}{w\;\left|\;Z{\left(w\right)}^{2}\right|}$$

**Figure 12:**
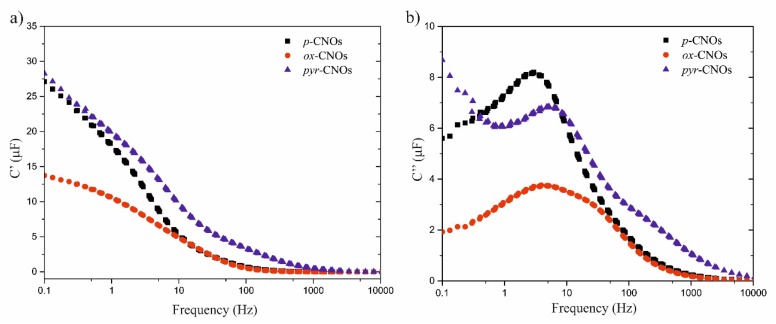
Dependence of the (**a**) real part of the capacitance and the (**b**) imaginary part of the capacitance on the frequency of the CNOs.

The dependence of the real and the imaginary capacitance on the frequency is shown in Figure 12a,b, respectively. The largest value of the real capacitance was obtained at low frequencies for the *pyr*-CNOs, which agreed with the CV measurements. In the *C’’* vs. log *f* plot, a maximum was observed, denoted as the relaxation frequency, *f_R_*, which is related to the time constant, *τ_R_*, indicating the time that was required to charge the capacitor through the resistor from an initial charge voltage of zero to approximately 63.2% of the value of an applied voltage [72]. The *τ_R_* values of each nanomaterial are indicated in Table 4, showing that the film charging process was relatively fast compared to the charging process of a traditional aluminum electrolytic capacitor and a carbon–carbon supercapacitor, which have *τ_R_* values of 126 and 7.94 µs [73], respectively, and Multiwalled carbon nanotubes (MWCT)-based capacitors, with a relaxation time of ~0.1 s [74]. This indicates that the CNOs in this study are the type that can be used for high power over short-period designs.

## 4. Conclusions

The functionalization of CNOs with pyrene moieties increases the stability of the resulting dispersions in methanol to more than two weeks, and this stability enhances the potential of using CNOs as a capacitor material. The porous morphology of the nanomaterials was corroborated by SEM. Though the strong oxidation of the *p*-CNOs, yielding *ox*-CNOs, decreased the ability of the CNOs to act as supercapacitor materials, subsequent amidation with pyrene hydrazide was effective, increasing the specific capacitance by 138% with respect to that of the pristine CNs. The EIS experiments suggest reducing both the resistance to the solution and the charge transfer in the functionalized nanostructure. This fact could be associated with the aggregation of pyrene groups, which could contribute to the formation of porous inter- and/or intraclusters. On the other hand, the faradaic reactions of the *pyr*-CNOs could also contribute to the specific capacitance through pseudocapacitive storage. The latter was corroborated by the deviation from the 90° pure-capacitor angle phase in the *pyr*-CNOs. These results indicate that π–π stacking functionalization is a good strategy for improving power characteristics for the development of electronic devices.

## Figures and Tables

**Table 1:** D and G band intensity ratios of the functionalized and non-functionalized CNOs.

Sample	υ_G_/cm^−1^ (FWHM)	υ_D_/cm^−1^ (FWHM)	υ_2D_/cm^−1^ (FWHM)	I_D_/I_G_	I_2D_/I_G_
*p*-CNOs	1583 (88.0)	1340 (106.0)	2675 (72.9)	1.08	0.24
*ox*-CNOs	1585 (74.6)	1340 (75.4)	2680 (80.0)	1.33	0.29
*pyr*-CNOs	1581 (81.6)	1348 (111.6)	2678 (62.2)	0.71	0.38

**Table 2:** The specific capacitance of the functionalized and non-functionalized CNOs based on the CV curve.

Sample	Specific Capacitance (Equation (2)) (F/g)	Specific Capacitance (Equation (3)) (F/g)
*p*-CNOs	36.0	26.1
*ox*-CNOs	29.3	19.8
*pyr*-CNOs	69.7	62.2

**Table 3:** Specific capacitance values obtained for the CNO materials.

Material	Specific Capacitance (F/g)	Reference
*oz*-CNOs	<43.4	[47]
CNOs/PDDA	9.26–33.41	[57]
CNOs/PEDOT:PSS	35.16–96.47	[58]
EQ *ox*-CNOs	42–99	[59]
*ox-*CNOs/C_60_-Pd	<280	[60]
CNOs/Chit	8.53–32.07	[57]
CNOs/RuO_2_*x*H_2_O	27–334	[21]
CNOs/Ppy	800	[61]
CNOs/Ni(OH)_2_	1225	[62]
N-CNOs	113	[63]
CNOs-ZnO/ZnO	125	[64]
CNOs/Mn_3_O_4_	195	[65]

**Table 4:** Electrochemical impedance spectroscopy (EIS) parameters that were determined for the GCE electrode that was modified with the functionalized and non-functionalized CNOs.

Sample	R_s_ (Ω)	Y_0_ (μF)	n	Z_W_ (Ω)	R_CT_ (Ω)	C_P_ (μF)	*ϕ* (°)	*τ_R_* (μs)
*p*-CNOs	63	291	0.91	354	52	41	80.6	9.36
*ox*-CNOs	91	137	0.93	130	143	154	77.9	7.18
*pyr*-CNOs	18	279	0.84	10^−12^	30.5	76	73.2	13.20
